# Hypoxic stress accelerates the propagation of pathological alpha‐synuclein and degeneration of dopaminergic neurons

**DOI:** 10.1111/cns.14055

**Published:** 2022-12-13

**Authors:** Mengyuan Guo, Weijin Liu, Hanjiang Luo, Qianqian Shao, Yuning Li, Yakun Gu, Yuying Guan, Wei Ma, Min Chen, Hui Yang, Xunming Ji, Jia Liu

**Affiliations:** ^1^ Beijing Institute of Brain Disorders, Laboratory of Brain Disorders, Ministry of Science and Technology, Collaborative Innovation Center for Brain Disorders, Beijing Advanced Innovation Center for Big Data‐based Precision Medicine Capital Medical University Beijing China; ^2^ Department of Neurobiology, School of Basic Medical Sciences Capital Medical University Beijing China; ^3^ School of Rehabilitation Medicine Capital Medical University Beijing China; ^4^ Neuroscience Laboratory Affiliated Hospital of Guilin Medical University Guangxi China; ^5^ Department of Neurosurgery, Xuanwu Hospital Capital Medical University Beijing China

**Keywords:** alpha‐synuclein, hypoxia, neurodegeneration, Parkinson's disease, propagation

## Abstract

**Aims:**

The etiology of Parkinson's disease (PD) is complex and the mechanism is unclear. It has become a top priority to find common factors that induce and affect PD pathology. We explored the key role of hypoxia in promoting the pathological propagation of α‐synuclein (α‐syn) and the progression of PD.

**Methods:**

We performed PD modeling by conducting intracranial stereotaxic surgery in the unilateral striatum of mice. We then measured protein aggregation in vitro. The rotarod and pole tests were employed next to measure the damage of the phenotype. Pathological deposition and autophagy were also observed by immunofluorescence staining and protein levels measured by western blotting.

**Results:**

We demonstrated that short‐term hypoxia activated phosphorylated (p)‐α‐syn in mice. We confirmed that p‐α‐syn was more readily formed aggregates than α‐syn in vitro. Furthermore, we found that hypoxia promoted the activation and propagation of endogenous α‐syn, contributing to the earlier degeneration of dopaminergic neurons in the substantia nigra and the deposition of p‐α‐syn in our animal model. Finally, autophagy inhibition contributed to the above pathologies.

**Conclusion:**

Hypoxia was shown to accelerate the pathological progression and damage phenotype in PD model mice. The results provided a promising research target for determining common interventions for PD in the future.

## Graphical abstract

Hypoxic stress specifically promotes α‐synuclein (α‐syn) aggregation and propagation by inducing the conversion of endogenous α‐syn into p‐α‐syn, thereby accelerating α‐syn prefabricated fibrils‐induced loss of dopaminergic neurons in the substantia nigra and PD‐like behavioral deficits in mice.

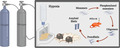

## 1 INTRODUCTION

Parkinson's disease (PD) is the second most common neurodegenerative disease in the world.^1^ It is a multi‐system disease affecting central and peripheral areas.^2^ The typical pathological features of PD are progressive degeneration and loss of dopaminergic neurons in the substantia nigra,^3^ accompanied by the formation of pathological inclusion bodies in the remaining neurons, namely Lewy bodies, is mainly composed of alpha‐synuclein (α‐syn).^4^ Although α‐syn has strong physiological functions under physiological conditions, it becomes a typical pathological feature of PD after undergoing a series of abnormal changes.^5^ Indeed, phosphorylated (p)‐α‐syn has been shown to be the main pathological form of α‐syn, and autopsy results of PD patients have indicated that 90% of the protein composition in Lewy bodies to be p‐α‐syn.^6^ P‐α‐syn contributes to aggregation of α‐syn in the form of monomers, dimers, oligomers, fibrils, and fibrous aggregates, which promote the transition of α‐syn from non‐toxic to toxic forms; and among these, α‐syn oligomers are considered to be the most neurotoxic form.^7^

Parkinson's disease is not a regional disease, but can progress in multiple systems, and as such, oligomeric α‐syn is thought to drive the progression of PD like a seed. The propagation of pathological α‐syn occurs in two forms: internal and trans‐neuronal.^8^ Braak initially hypothesized and demonstrated that α‐syn pathology originated in the gastrointestinal tract and subsequently manifested in the ventral midbrain to target dopaminergic neurons^9^; subsequently, more literature began to review the close connection between the gut and PD.^10^ α‐syn pre‐formed fibrils (α‐syn PFFs, hereinafter referred to as PFFs) synthesized by recombinant in vitro techniques are similar to in vivo the α‐syn aggregates and widely used to study PD pathology.^11^ Indeed, PFFs injected into the striatum of mice can induce neuronal propagation of pathological α‐syn in multiple brain regions and induce midbrain dopaminergic neuron death.^12^ Furthermore, injection of PFFs into the gastric pylorus or duodenum revealed retrograde multisynaptic diffusion of α‐syn along the vagus nerve, with eventual aggregation in the substantia nigra region.^13^ Therefore, α‐syn pathology and its propagation play a significant role in PD pathogenesis, but the underlying driving factors are still unclear.

The complex etiology of PD makes it extremely difficult to study and identify targets for intervention. However, there are potential common characteristics which may aid in the possible intervention of PD.^14^ The etiology of PD can be divided into two categories: inadequate oxygen intake and oxygen utilization disorders. Secondary trauma to traumatic brain injury can cause hypoxia to the brain,^15^, ^16^, ^17^ resulting inadequate oxygen intake. Furthermore, overexpression of *SNCA* causes mitochondrial fracture and damages mitochondrial function, leading to decreased respiratory function. PD risk factors such as rotenone^18^ and paraquat^19^ can also cause mitochondrial dysfunction. In addition, there is a clear etiology associated with hypoxia such as CO poisoning,^20^ stroke and arteriosclerosis,^21^ which can also cause secondary PD‐related lesions. Precisely because of the critical role of hypoxia in the pathogenesis of PD, modulation of hypoxic response has also become a potential treatment strategy for PD.^22^, ^23^ Hypoxia seems to be subtly associated with α‐syn pathology, with increased total α‐syn and p‐α‐syn expression detected both in clinical patients with obstructive sleep apnea^24^ and in animal models of arterial injury.^25^ Therefore, hypoxia is closely related to the pathogenesis of PD, but the role of hypoxia on the pathological progression of α‐syn remains unclear.

In this study, we aimed to explore whether hypoxia could be a critical contributor in promoting α‐syn pathology and its propagation. We found that short‐term hypoxia can convert α‐syn into p‐α ‐syn in the brain. Using in vitro experiments, we confirmed that p‐α‐syn was triggered by PFFs to form aggregates more quickly and readily than α‐syn. We also found that hypoxia facilitated faster and more sensitive propagation of α‐syn pathology from the striatum to midbrain, and induced dopaminergic neuron loss and PD‐like motor symptoms in an animal model.

## 2 MATERIALS AND METHODS

### 2.1 Animals

Adult male C57BL mice aged 2–3 months were used in this study. All animals were kept under ventilated room temperature conditions with free access to food and water, regular replacement of fresh supplies, and a 12‐h light–dark cycle. Our study was approved by the Ethics Committee of Beijing Institute of Brain Disorders in Capital Medical University. The animal experiments were conducted in accordance with the in vivo experiments (ARRIVE) guidelines for National Institutes of Health research.^26^ In the rotarod test, animals that cling to the track on all fours rather than running freely were excluded. In the pole test, animals that are afraid of heights and do not advance are excluded.

### 2.2 Hypoxia treatment

All mice exposed to hypoxic conditions completed the hypoxic treatment in closed controlled hypoxic chambers (Ningbo Hua Yi Ning Chuang, Ningbo, China, Attendor Pro). The hypoxic chambers can accurately set the hypoxic concentration and meet the persistent hypoxic mode. In this experiment, 13% O_2_ was used for 0 day, 1 day, 3 days, 7 days and 14 days (Con, H1d, H3d, H7d, H14d). Short‐term hypoxia was considered to be within 3 days, and prolonged to 7 days or more is considered as long‐term hypoxia. The hypoxic chambers were opened every 3 days at the same time to replenish fresh food and water, and replaced dry adsorbent particles to ensure a clean environment for animals.

### 2.3 Preparation of α‐syn monomers

Recombinant human α‐syn was purified by referring to previously published methods.^11^ In brief, the resuspended competent cells were plated on preheated solid medium and incubated for 12–16 h at 37°C to form colonies and then for 12 h at 37°C and 220 rpm. The above bacterial liquid and medium were allowed to expand at a ratio of 1:100 for 1.5–2 h at 37°C and 220 rpm. IPTG (Sigma, 16758) at a final concentration of 0.1 mM was added to the above bacterial solution for 4 h at 37°C and 260 rpm. The solution was then centrifuged (Eppendorf, 5430R) for 10 min at 8000 g and 4°C to collect the precipitated bacteria. Protein was enriched with glutathione sepharose beads (GE Health, 17–0756‐01) for 4–6 h at 4°C. The protein tag was excised and then collected the target protein.

### 2.4 Catalytic phosphorylation of α‐syn

Approximately 400 μl of α‐syn (2 mg/ml) was mixed 24 μl of PLK3 (Thermo, PV3812) and 8 μl of ATP (Sigma, A26209‐1G, 100 mM) into a 1.5 ml EP tube and incubated for 3 h at 30°C, after which 48 μl EDTA‐NA2 (Solarbio, E8030), at a final concentration of 25 mM, was added.

### 2.5 α‐syn PFFs preparation

The concentration of α‐syn was adjusted to 2 mg/ml, and 120 μl α‐syn was placed in an airtight 200 μl PCR tube (AXYGEN, PCR‐0208‐C). PFFs were prepared by shaking on a constant temperature shaker (Heidolph, Titramax 1000) for 7 days at 37°C and 1000 rpm.

### 2.6 ThT assay

The thioflavin T (ThT) assay was performed as previously described^27^ to confirm the presence of beta‐sheet structures. Briefly, 5 μl of α‐syn (2 mg/ml) and 95 μl of ThT (20 μM) were mixed and incubated for 30 min at room temperature in the dark, and then scanned with a multifunctional microplate reader (Molecular Devices, Spectra Max i3x). The microplate reader parameters were set to E‐440 nm and F‐480 nm.

### 2.7 Electron microscopy experiment

The sample concentration for electron microscopy was 20 μg/ml and diluted with PBS solution, about 50ul was needed. Then, a 200‐mesh carbon‐coated copper grid (Beijing Zhongjingkeyi Technology, BZ10022a) was levitated upside down on the sample droplet for 6 min. The incomplete dry copper mesh stained with the sample was inverted on the drop with negative dye solution, negative dye for 3 min. After the mesh was completely dried, the samples were observed under the microscope. The Hitachi HT7700 transmission electron microscope was used for observation.

### 2.8 Preparation of seeds

The seed concentration of PFFs used was 2 mg/ml. It has been shown that PFFs need to be broken into short fibrils <100 nm and, preferably ≤50 nm, by sonication to become pathogenic.^27^ Therefore, the sonicator (SCIENTZ, JY92‐II) was used with the following parameters: 25 W, 1 s on and 1 s off, for a total of 1 min. In addition, two parameters with another sonicator (SCIENTZ, 950E) were set as control, respectively 60 W and 90 W.

### 2.9 Aggregation of α‐syn in vitro

As described in the previous literature,^28^ both in vitro and in vivo studies have shown that the formation of amyloid fibers can be accelerated by the addition of prefabricated aggregates. PFFs were used as the trigger reagent, and α‐syn and p‐α‐syn as the trigger objects. PFFs (1 μM) and the α‐syn or p‐α‐syn solution were mixed at three different molar ratios: low (25:0.25), middle (25:0.5), and high (25:1). The mixture was carried out in 1.5 ml EP tubes (AXYGEN, McT‐150‐c) at 37°C with continuous shaking (1000 rpm). Sample mixtures were retained at each time point to carry out the above ThT assay to detect α‐syn aggregation. The experiment had six groups, including α‐syn low, α‐syn middle, α‐syn high, p‐α‐syn low, p‐α‐syn middle, and p‐α‐syn high. Samples were collected at four time points: 0, 1, 4, and 8 days.

### 2.10 Striatal stereotactic surgery

Unilateral striatal stereolocalization injection was performed. The injection position was determined by a stereotaxic instrument (RWD, 68018) with the following coordinates: A/P: −0.1 mm, M/L: −2 mm, D/V: −2.8 mm.

### 2.11 Motor behavior tests

The rotarod test used a rotarod meter (Panlab, LE8205) that can control and record speed and time (0–40 rpm with an acceleration time of 300 s). The pole test was carried out on a long rod about 48 cm in length. The mice were placed on a ball at the top of a wooden pole, and the time it took them to climb from the top to the bottom was recorded.

### 2.12 Gastrointestinal behavior tests

Two tests were used to measure bowel habits, including fecal water content and intestinal transit. Higher water content and transit ratio mean better conditions. Fecal particles were collected for 2 h. Using a previously reported calculation method,^29^ fecal water content (%) was determined by using the equation: (wet weight – dry weight)/wet weight × 100. The mice were orally administered 150 μl of the above paste as previously reported,^30^ named gavage treatment. The formula is: travel distance of charcoal × 100/length of small intestine (from pylorus to ileocecal region) = % Intestinal transit.

### 2.13 Immunohistochemical staining

Tissue paraffin slices were dewaxed in water and slices placed in box filled with 1 × citrate antigen repair buffer (pH 6.0) in a microwave oven for antigen retrieval. Next, the primary antibody (TH, Servicebio, GB12181, 1:1500) was added to the sections and incubated overnight.

### 2.14 Immunofluorescence staining

The primary antibodies used were p‐α‐syn (wako, 1: 1 W), TH (Servicebio, GB12181, 1:1500), MAP2 (Servicebio, GB11128‐2, 1:500), and P62 (sigma, P0067, 1:250). Cell nuclei were counterstained with DAPI. Four secondary antibodies: Alexa Fluor™ 488 Goat anti‐Mouse IgG (H + L) (Invitrogen, A11029), Alexa Fluor™ 594 Goat anti‐mouse IgG (H + L) (Invitrogen, A11032), Alexa Fluor™ 488 Goat anti‐rabbit IgG (H + L) (Invitrogen, A11034), and Alexa Fluor™ 594 Goat anti‐rabbit IgG (H + L) (Invitrogen, A11037).

### 2.15 Western blotting

The antibodies used were: β‐actin (HuaBio, EM21002, 1:10000–25,000), α‐syn (BD, 610787, 1:500), p‐α‐syn (CST, 23706, 1:1000), and LC3 (sigma, L7543, 1:1000). The secondary antibodies were: IRDye 680RD goat anti‐mouse IgG (H + L) (Licor, 926‐68070), IRDye 800CW goat anti‐mouse IgG (H + L) (Licor, 926‐32210), IRDye 680RD goat anti‐rabbit IgG (H + L) (Licor, 926‐68071), and IRDye 800CW goat anti‐rabbit IgG (H + L) (Licor, 926‐32211). All original blot images were available in Appendix S1.

### 2.16 Statistical analysis

All raw data were recorded and saved using Excel, and GraphPad Prism 9.3.1 software was used for statistics and analysis of raw data. Data are expressed as the mean ± SEM, and *p* < 0.05 was considered statistically significant. The Shapiro–Wilk normality test was used for checking the normality of variables. The unpaired *t*‐test (two‐tailed) was performed to two groups of normal distribution and equal variances data. Otherwise, nonparametric tests were used. For multiple groups, one/two‐way analysis of variance (ANOVA) followed by the Tukey post hoc test put into effect. Image data were analyzed by ImageJ software.

## 3 RESULTS

### 3.1 Short‐term hypoxia promoted non‐selective conversion of α‐syn into p‐α‐syn in C57BL mice

**FIGURE 1:**
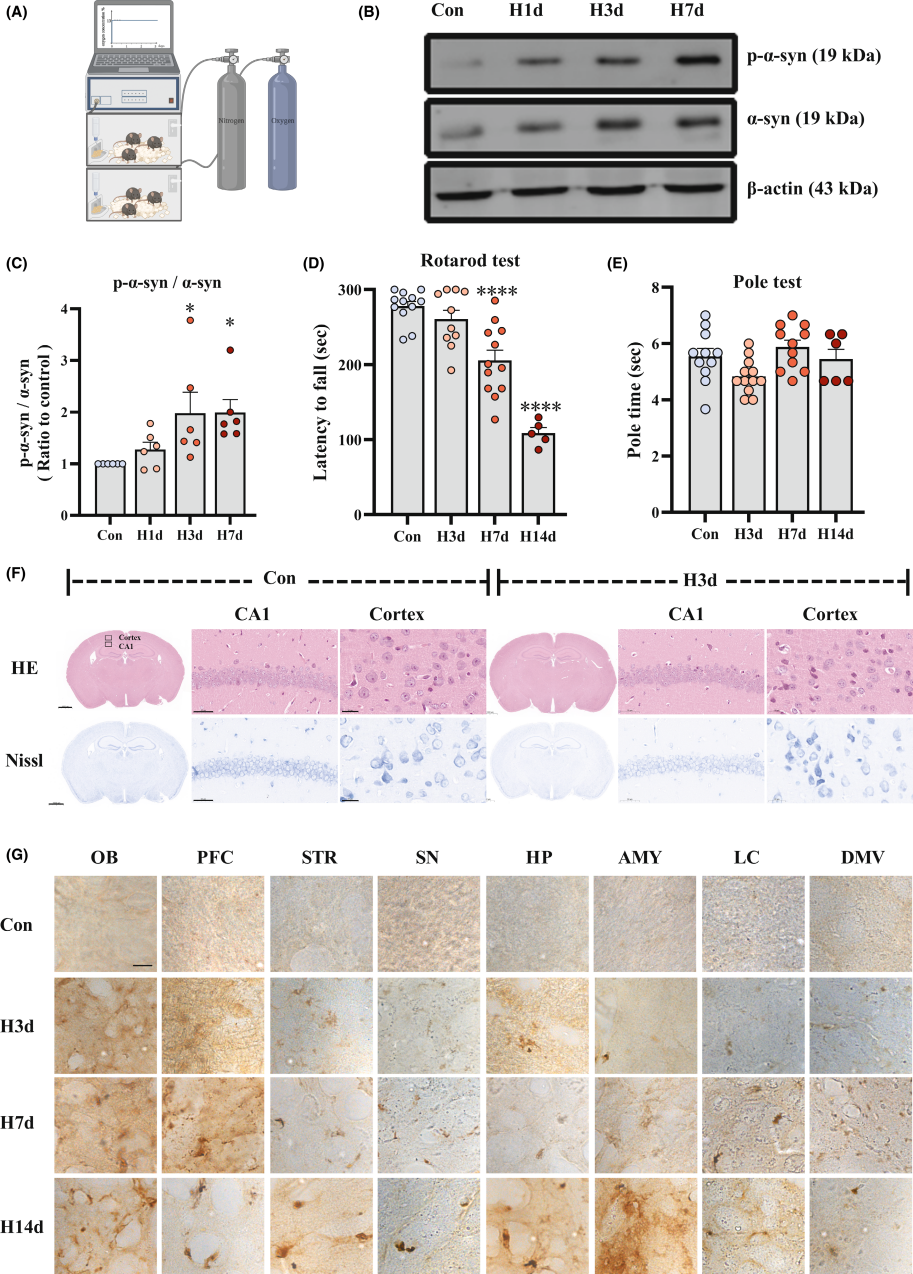
Short‐term hypoxia promoted non‐selective conversion of α‐syn into p‐α‐syn in the brains of model mice. (A) Specific hypoxia patterns. Mice were placed in hypoxic chambers with an O_2_ concentration of 13%. Constant levels of nitrogen and oxygen were maintained to achieve persistent hypoxia. (B) Mice were continuously treated with hypoxia for 0, 1, 3, and 7 days (Con, H1d, H3d, H7d). The levels of p‐α‐syn and α‐syn in cortical protein lysates were detected by western blot using β‐actin as the internal reference. (C) Statistical analysis of western blot was performed by homogenization which means set the value of Con group as 1. (D–E) Mice were continuously treated with hypoxia for 0, 3, 7, and 14 days (Con, H3d, H7d, H14d), and behavioral tests were performed at different time points. (D) Detection and statistical analysis of rotarod test in mice of each group. (E) Detection and statistical analysis of pole test in mice of each group. (F) H&E and Nissl staining were used to evaluate the neuronal arrangement in the cortex and hippocampus. (G) The levels of p‐α‐syn in the eight brain regions [olfactory bulb (OB), prefrontal cortex (PFC), striatum (STR), hippocampus (HP), amygdala (AMY), substantia nigra (SN), locus coeruleus (LC), and dorsal nucleus of the vagus nerve (DMV)] of the Con, H3d, H7d, and H14d groups were detected by immunohistochemistry: Amygdaloid nucleus, AMY; dorsal motor nucleus of the vagus, DMV; hippocampus, HP; locus coeruleus, LC; olfactory bulb, OB; prefrontal cortex, PFC; striatum, STR; substantia nigra, SN. Data are expressed as the mean ± SEM (one‐way ANOVA). *p < 0.05, ****p < 0.0001 vs. control (Con), *n* = 5–12. Bar = 1000 μm, 50 μm, 20 μm in F, Bar = 10 μm in G.

To investigate whether hypoxia causes abnormal changes in α‐syn, we first identified a reliable hypoxic pattern, as shown in Figure 1A. We established hypoxic models by continuously exposing mice to 13% O_2_ for 0, 1, 3, and 7 days (Con, H1d, H3d, H7d). Western blot of mouse brain tissue homogenates (Figure 1B,C) showed that p‐α‐syn increased significantly on day 3 of hypoxia compared with the Con group. To determine whether hypoxia causes behavioral changes in mice, we performed behavioral tests on different groups of mice. It was found (Figure 1D,E) that after 3 days of hypoxia did not alter the behavioral performance of mice in rotarod and pole tests. At the same time, Nissl staining also showed no neurological damage after 3 days of hypoxia (Figure 1F). To observe the ability of hypoxia to induce α‐syn pathology, we performed p‐α‐syn immunohistochemical staining on the brain tissues of hypoxic mice. The results (Figure 1G) showed that after 3 days of hypoxia, p‐α‐syn levels increased in all brain regions, while longer periods of hypoxia promoted p‐α‐syn aggregation; however, abnormal changes in α‐syn induced by hypoxia were not regionally selective, there was an increase in all regions. The results suggested that 3‐day hypoxic treatment induced α‐syn pathology in the brain to a large extent, but there was no evidence of neurological damage or behavioral abnormalities, and no significant pathological deposition of p‐α‐syn. Therefore, in the subsequent experiments, 3‐day hypoxic treatment was used to study the effect of hypoxia on the pathologic progression of PFF‐induced α‐syn.

### 3.2 PFFs triggered p‐α‐syn but not α‐syn to form aggregates more quickly and readily in vitro

**FIGURE 2:**
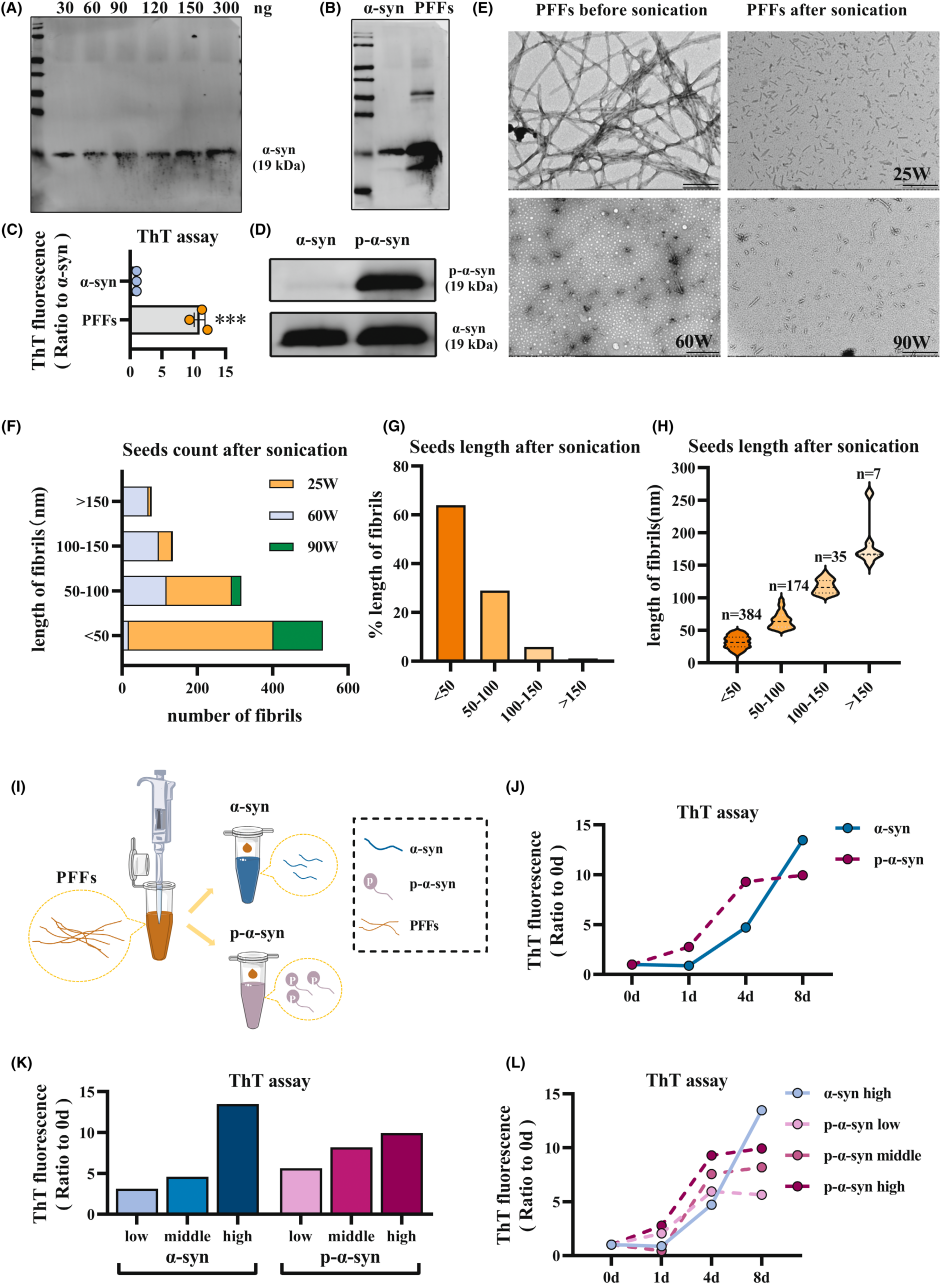
α‐syn PFFs triggered p‐α‐syn but not α‐syn to form aggregates more quickly and readily in vitro. (A) Western blot was used to detect the expression level of α‐syn in gradient doses. (B) The expression of PFFs was verified by western blot. (C) ThT assay was used to detect the level of β‐sheets in α‐syn and PFFs. (D) α‐syn was used as a control, and the successful conversion of α‐syn to p‐α‐syn was verified by western blot. (E) The aggregated PFFs without any treatment (pre‐sonication) and the short PFF fibrils under different parameters were both observed by transmission electron microscopy (post‐sonication). (F–H) The number and size of short PFF fibrils after sonication treatment were in line with the pathogenic requirements. (F) Number and interval distribution of PFF fibrils treated with various sonication parameters. (G) Under the parameter of 25 W, the proportion of PFF fibrils with different lengths. (H) PFF fibrils of different lengths were counted under 25 W. (I–L) Protein aggregation assay in vitro. (I) Schematic diagram of the experiment. PFFs were added proportionally to the tube containing α‐syn and p‐α‐syn to trigger aggregation. (J) The aggregation experiment lasted 0–8 days (0d, 1d, 4d, 8d), and ThT assay was used to detect the aggregation trend of α‐syn and p‐α‐syn. (K) In the aggregation experiment, six groups (α‐syn low, α‐syn middle, α‐syn high, p‐α‐syn low, p‐α‐syn middle, p‐α‐syn high) were set up. ThT assay was used to detect the level of β‐sheets in each group after 8 days. (L) The group named α‐syn high was used as a control to compare the trend of the three p‐α‐syn‐concentration groups. Data are expressed as the mean ± SEM (*t* test). ***p < 0.001 versus α‐syn, *n* = 3. Bar = 500 nm, 200 nm in E.

Injection of PFFs to simulate PD is a well‐established modality, which can specifically mediate selective propagation of α‐syn pathology in specific tissues or brain regions.^31^ We first constructed PFFs and α‐syn monomers in vitro (Figure 2A). Western blot results showed that compared with α‐syn monomers, PFFs were composed of a number of high‐molecular weight proteins (Figure 2B). ThT assay was used to confirm the presence of β‐sheet in PFFs,^32^ which is an important process for α‐syn aggregation.^33^ The results showed that compared with α‐syn monomers, the ThT fluorescence value of PFFs increased by 10 times (Figure 2C). Electron microscope experiments confirmed that α‐syn in PFFs aggregated into long fibers larger than 200 nm (Figure 2E), which verified the successful preparation of PFFs. The length of the fibrils was greatly important for the pathogenicity of PFFs.^27^ Three sonication parameters were examined in this experiment. The results in Figure 2F show that PFFs under the parameter of 25 W were fragmented to a large extent and that the number of fibrils below 50 nm was >60%, which met the requirements for pathogenicity (Figure 2G,H).

We found that hypoxia can induce α‐syn to form p‐α‐syn, so we suspected that activated p‐α‐syn would likely promote rapid pathological propagation. Purified α‐syn protein was phosphorylated in vitro by PLK3 kinase,^34^ which was confirmed by western blotting (Figure 2D). The in vitro experiments are shown in a pattern diagram (Figure 2I). In order to observe the aggregation rate and sensitivity of α‐syn and p‐α‐syn, prepared PFFs were added at a 1:1 ratio with the α‐syn and p‐α‐syn mixture (Figure 2I). Firstly, PFFs (1 μM) induction concentration commonly used in literature was taken, and the same amount of PFFs was added into the two test tubes respectively, and ThT was monitored for 8 days. The results showed that p‐α‐syn formed β‐sheets more quickly than α‐syn, reaching a peak at about 4 days and then stabilizing, which suggested that the aggregation of p‐α‐syn occurs at a quicker rate than α‐syn (Figure 2J). In order to compare the sensitivity of α‐syn and p‐α‐syn in the formation of aggregates triggered by PFFs, three different PFFs concentrations were used. The results of ThT assay showed that the low‐ and middle doses of PFFs did not induce effective α‐syn aggregation but did induce p‐α‐syn aggregation, as compared with high‐dose PFFs (Figure 2K). Moreover, p‐α‐syn aggregation triggered by PFFs at different doses showed a similar trend, indicating faster aggregation that induced and maintained high levels of β‐sheets (Figure 2L). These results suggest that p‐α‐syn aggregates more quickly and sensitively than α‐syn, and can be triggered by lower doses of PFFs.

### 3.3 Short‐term hypoxia accelerated the PFF‐induced damage phenotype in PD.

**FIGURE 3:**
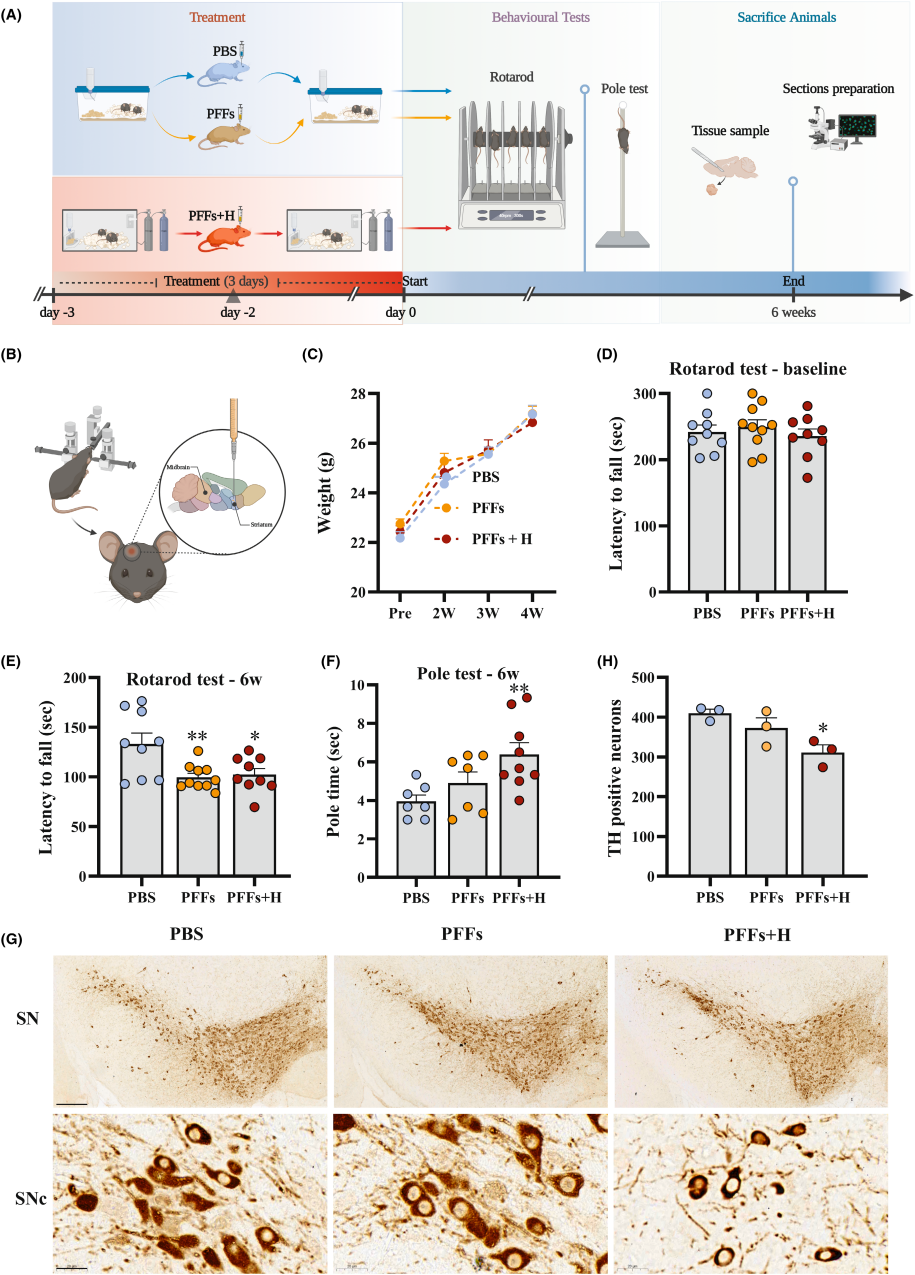
Short‐term hypoxia accelerated PFF‐induced damage phenotype in PD. (A) The timeline. The experimental animals were divided into three groups: the group injected with 2 μl PBS solution alone was named PBS, the group injected with 5 μg of PFFs solution alone was named PFFs, and the group injected with 5ug PFFs solution combined with 3‐day hypoxia treatment was named PFFs + H. Three groups of animals were injected with PBS/PFFs at the same time point on the first day. In other words, animals in the PFFs + H group were put into the hypoxic chambers for 24 h in order to activate α‐syn into p‐α‐syn to a certain extent by hypoxia. Then each animal in the PFFs + H group was briefly removed from the hypoxic chambers and injected with PFFs. At the same time, animals in the PBS and PFFs groups raised in animal houses were injected with either PBS or PFFs. After injection, the animals in the PFFs + H group were placed back into the hypoxic chambers for 48 h. Animals in the PBS and PFFs groups were continued to be kept in the animal house for 48 h. At the end of this 3‐day hypoxic period, all the animals were kept in the animal house for observation. The end point of observation was at 6 weeks after the initial injection. (B) Stereotactic injection of PFFs in the unilateral striatum of mice. (C) Body weight of animals in each group during modeling. (D) The baseline of rotarod test and statistical analysis of each group of mice. (E) Rotarod test and statistical analysis of each group of mice at 6 weeks. (F) Pole test and statistical analysis of each group of mice at 6 weeks. (G) TH staining was used to evaluate the loss of dopaminergic neurons in substantia nigra pars compacta. (H) Statistical analysis of TH staining. Data are expressed as the mean ± SEM (one‐way ANOVA). *p < 0.05, **p < 0.01 versus PBS, *n* = 3–10. Bar = 200 μm, 20 μm in F.

The initial findings indicated that 3 days of hypoxia can induce the conversion of a‐syn to p‐a‐syn in the brain, and that p‐α‐syn can be triggered by PFFs more quickly and sensitively than α‐syn in vitro; therefore, we next used a mouse model of PD to investigate whether hypoxia contributes to PFF‐induced α‐syn pathological propagation and PD progression. PFFs were fragmented fibrils <50 nm by sonication (Figure 2E). C57BL mice aged 2–3 months were divided into three experimental groups (PBS group, PFFs group, and PFFs + H group) and mice in each received a stereotaxic injection of PFFs into the striatum and subsequent hypoxia treatment (Figure 3A,B). Six weeks after modeling, mice in each group were tested by rotarod and pole tests. During the period, animals gained weight normally (Figure 3C). Before modeling, mice in each group were tested to get the baseline data, which showed no difference (Figure 3D). Rotarod test results showed that mice in the PFFs group and mice in the PFFs + H group showed motor behavior impairment at 6 weeks after injection (Figure 3E). Pole test results showed that mice in the PFFs + H group had impaired motor ability compared with mice in the PFFs group alone (Figure 3F). The results showed that hypoxia combined with PFF injection could aggravate motor symptoms in the model mice. We used TH to label dopaminergic neurons to observe the number of TH‐positive neurons in each group of mice. TH immunohistochemical staining of the substantia nigra showed that mice exposed to hypoxia and injected with PFFs had a significantly lower number TH‐positive neurons compared with the PBS group (Figure 3G,H). These results suggest that short‐term hypoxia stress accelerates the behavioral decline and dopaminergic neuron loss in PFF‐induced model mice.

### 3.4 Short‐term hypoxia accelerated p‐α‐syn specific deposition by inducing autophagic flux inhibition in the midbrain of PFF‐induced mice

**FIGURE 4:**
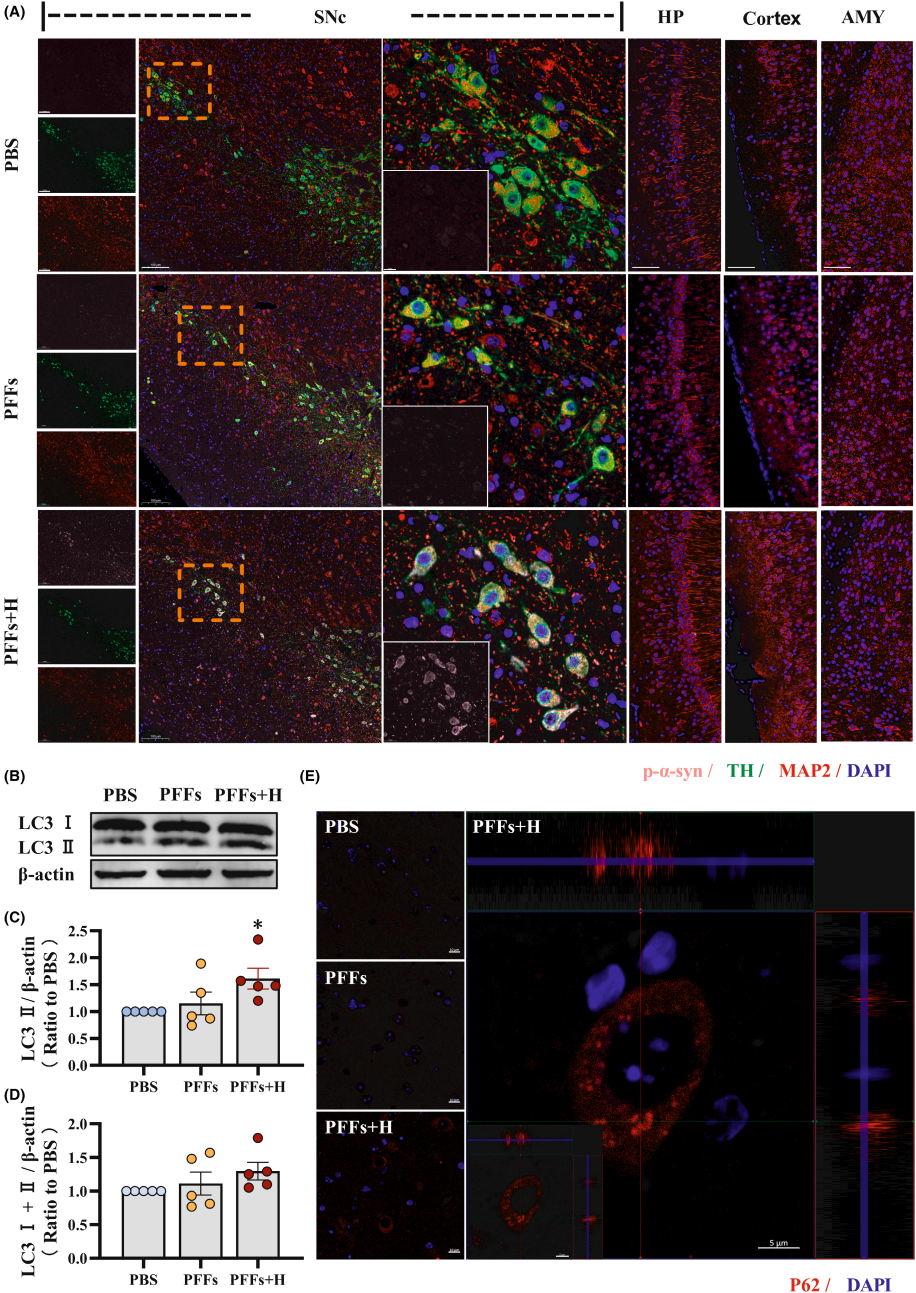
Short‐term hypoxia accelerated p‐α‐syn specific deposition by inducing autophagic flux inhibition in the midbrain of PFF‐induced mice. (A) Immunofluorescence results. Three antibodies, p‐α‐syn, TH, and MAP2, were used for co‐labeling experiments, and the neuronal loss and pathological deposition in neurons in each group were detected. Bar = 100 μm, 50 μm. (B) Western blotting was used to detect LC3 in midbrain protein lysates. (C) The change trend of LC3II among the three groups. (D) The statistics of total LC3. (E) The Immunofluorescence result of P62 among the three groups. Data are expressed as the mean ± SEM (one‐way ANOVA). *p < 0.05 versus PBS, *n* = 5. Bar = 10 μm, 5 μm in E.

We have previously shown that hypoxia promotes conversion of α‐syn into p‐α‐syn, a pathogenic form that promotes pathology and propagation of α‐syn and exacerbates PD progression. Meanwhile, we have found that hypoxic stress accelerated the behavioral decline and dopaminergic neuron loss in PFFs‐induced model mice and further observed the pathological changes of p‐α‐syn in this model. We next performed triple immunofluorescence staining of p‐α‐syn, TH and the neuronal marker MAP2 in the substantia nigra of each group of model mice to observe the relationship between the pathological changes of p‐α‐syn and the damage of dopaminergic neurons (Figure 4A). The results showed that the hypoxia treatment group induced more obvious p‐α‐syn staining with partial aggregation. Meanwhile, the number of TH‐positive neurons decreased, and the cell body of neurons was pyknotic and the neurites ruptured. As a control, p‐α‐syn was also detected by immunofluorescence in other brain regions, including the hippocampus (HP), cortex, and amygdala (AMY), with no p‐α‐syn staining present. These results suggest that hypoxia exacerbates the transmission of α‐syn pathology and dopaminergic neuron loss in the substantia nigra specifically by promoting the conversion of α‐syn into p‐α‐syn, thereby promoting disease progression in the model mice.

In the above studies, we found that hypoxia accelerated the formation of p‐α‐syn aggregates. The formation of p‐α‐syn aggregates is mainly affected by two factors. The first is that hypoxia was found to induce α‐syn into p‐α‐syn. The next is whether p‐α‐syn was able to be cleared successfully. In neurons, autophagy is the most important way to clear toxic proteins.^35^ Therefore, we further conducted western blotting on midbrain tissue of model mice to explore the changes of autophagy marker microtubule‐associated protein LC3. The results showed that the levels of LC3 II increased significantly the PFFs + H group compared with the PBS group. (Figure 4B–D). There were two possibilities for the increase of LC3. First, LC3 levels may have increased due to autophagic induction or LC3 degradation was blocked due to autophagic flow inhibition. We examined total LC3 levels, but found no statistically significant change in total LC3 level between the PFFs + H group and PBS group (Figure 4D). We therefore speculated that there was autophagic flux inhibition in the PFFs + H group. We further detected the level of P62 in dopaminergic neurons in the substantia nigra of each group of mice by immunofluorescence staining. The results showed that P62 was widely distributed in the cells in the PBS and PFFs groups, whereas P62 was clustered in a puncta formation in dopaminergic neurons of the PFFs + H group (Figure 4E). The results further suggested that autophagic flow was blocked. In summary, we found that hypoxia induced autophagic flow inhibition, which accelerated PFF‐induced p‐α‐syn aggregation and dopaminergic neuron loss.

### 3.5 Hypoxia alone affected the gastrointestinal behavior and p‐α‐syn deposition in the small intestine of model mice in a time‐dependent manner

**FIGURE 5:**
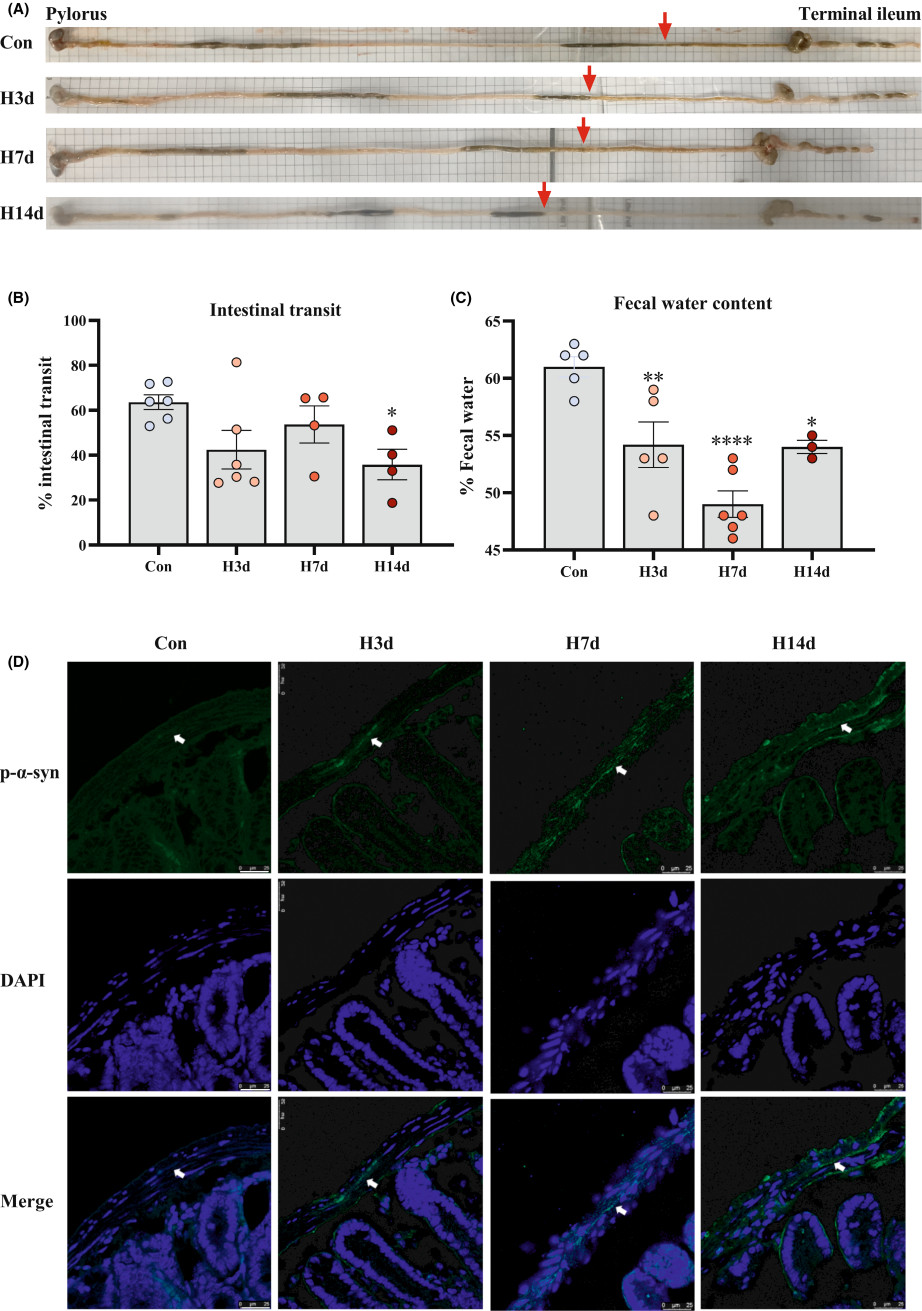
Hypoxia alone affected the gastrointestinal behavior and p‐α‐syn deposition in the small intestine of model mice in a time‐dependent manner. (A) Photos taken from mice of each group after gavage as mentioned in the methods. (B) Intestinal transit of mice treated with hypoxia for 0, 3, 7, and 14 days (Con, H3d, H7d, H14d). (C) Fecal water content of mice treated with hypoxia for 0, 3, 7, and 14 days (Con, H3d, H7d, H14d). (D) The pathological deposition of p‐α‐syn in the small intestine was detected by immunofluorescence. Data are expressed as the mean ± SEM (one‐way ANOVA). *p < 0.05, **p < 0.01, ****p < 0.0001 versus Con, *n* = 3–6. Bar = 25 μm in D.

As mentioned above, some studies have suggested that pathological aggregation and retrograde transmission of α‐syn may occur early in the intestinal tract of PD patients in the premorbid stage.^9^ We explored this possibility in a simple model of persistent hypoxia. Models mice were subjected a hypoxic conditions (O_2_ concentration of 13%) for 0, 3, 7, and 14 days. As shown in Figure 5A, samples were taken after gavage treatment with paste mentioned in the methods. The gastrointestinal behavior test suggested that long‐term hypoxia, such as 14‐day treatment, would prolong the gastrointestinal transport time of mice (Figure 5A,B). Meanwhile, persistent hypoxia led to a reduction of fecal water content in mice, with the most obvious change occurring at 7 days (Figure 5C). In addition, gastrointestinal behavioral changes were also observed after 3 days of hypoxia. Immunofluorescence staining of intestinal sections showed that p‐α‐syn deposition was obvious in the H7d group and weaker in the H3d group (Figure 5D). These results suggest that short‐term hypoxia can also trigger intestinal pathological deposition of α‐syn, which to some extent provides a feasibility to explore the relationship between hypoxia and the intestinal origin of PD.

## 4 DISCUSSION

In this study, we found that chronic short‐term hypoxia induced the conversion of α‐syn into p‐α‐syn in the brains of model mice. Compared with α‐syn, p‐α‐syn aggregation induced by PFFs was faster and occurred more readily, which indicates a possible mechanism for the spread of α‐syn pathology in PD. In our model mice, we found that hypoxia promoted the formation of PFF‐induced α‐syn pathology and its propagation from the striatum to midbrain, which accelerated dopaminergic neuron loss and behavioral impairments. The results provide a new theoretical basis for blocking the pathological progression of PD with hypoxia as the target.

Our previous study found that hypoxia is a common factor of various pathogenic factors of PD.^36^ It is a new perspective to interpret PD from the perspective of hypoxia. The results of epidemiological studies have shown that aging is an important factor in increasing the incidence of PD, and that the incidence of PD increases 5–10 times after the age of 65.^37^ Exposure to environmental particulate matter has also been shown to be positively associated with PD.^38^ Interestingly, up to 45% of people with severe brain trauma experience hypoxia^15^ and previous traumatic brain injury is associated with an increased risk of PD, the risk of which is positively correlated with the degree of injury.^39^ Previous studies have shown that the modeling cycle of PD caused by injection of PFFs alone is long and the pathological propagation slow, with effective pathological propagation only being completed after at least 3 months.^40^ Indeed, a pathological increase in α‐syn in the substantia nigra was observed 3 months after injection of 5 μg of PFFs into the dorsal striatum of mice and neuronal death was observed at 6 months after injection.^41^ Another study indicated that after injection of 5 μg of PFFs into the unilateral striatum of mice, loss of dopaminergic neurons was detected and p‐α‐syn staining co‐localized TH in the substantia nigra.^42^ Our results showed that short‐term hypoxia stimulated and even accelerated PD‐like progression, with concurrent p‐α‐syn aggregation. At the same time, the degree and duration of hypoxia affected the pathological results. Short‐term hypoxia alone induced α‐syn formation into p‐α‐syn and long‐term hypoxia induced pathological aggregation and accumulation of p‐α‐syn in multiple brain regions, including the olfactory bulbs, midbrain, and hippocampus. These results showed that the pathological changes of α‐syn induced by hypoxia alone were widespread and had were not region specific. Our results also indicated hypoxia, regardless of duration, induced α‐syn to form p‐α‐syn, which not only increased significantly in the presence of PFFs but also presented as insoluble aggregates. Previous literature has confirmed that ischemia and hypoxia can induce α‐syn pathology. For example, transient MCAO ischemia^43^ resulted in increased cortical α‐syn levels.^44^ Furthermore, interference with α‐syn by siRNA therapy has been shown to be effective in mice with short‐term ischemia, suggesting that α‐syn pathology may be involved in ischemia‐hypoxia‐induced injury. Interestingly, the protective effect of this intervention was much lower in older mice than in younger mice, possibly because there was more accumulation of α‐syn in the brains of older mice.^45^

Our study demonstrated that conversion of α‐syn to p‐α‐syn increased in response to hypoxic stimulation. In recent years, a large number of studies have confirmed the intestinal origin theory of PD, suggesting that α‐syn originates first in the enteric nervous system and then extends up into the central nervous system. We suspect that hypoxia may play an integral role in this process. Studies have shown that hypoxia and hypoxia signaling pathways play an important role in intestinal lesions.^46^ Hypoxia in the gut can directly trigger inflammatory bowel disease.^47^ Pathological aggregates of α‐syn were also detected in the gastrointestinal submucosa of patients with enteritis.^48^ Multiple cohorts suggest that patients with inflammatory bowel disease have a 20% higher risk of developing PD in later life.^49^ In conclusion, hypoxia probably plays a facilitator role in the origin and progression of PD. We highlight the important role of hypoxia in the pathological propagation of α‐syn and the progression of PD, which provides new possibilities for finding reliable targets to interfere with PD process in the future. However, a model of hypoxia involving the gut brain axis has not been clarified yet, and further follow‐up studies are needed.

We found that hypoxia accelerated PFF‐induced dopaminergic neuron loss and PD phenotypic emergence. The essential factor was likely the increased p‐α‐syn deposition found in the brains of model mice. The increase of p‐α‐syn deposition depended on two main factors. First, hypoxia induced the conversion of α‐syn to p‐α‐syn, which was confirmed by western blot. Meanwhile, it has been shown that phosphorylation modification at Ser129 is the main modification that promotes α‐syn aggregation and induces α‐syn pathology. In addition, p‐α‐syn is more likely to form aggregates than α‐syn,^50^, ^51^ which we confirmed here in vitro. Second, western blotting and immunofluorescence results showed that the protein levels of LC3 and P62 were altered in model mice and that the pathological deposition of p‐α‐syn increased. These results showed that hypoxia caused the blockage of autophagic flow and as a result p‐α‐syn was not degraded, which further aggravated the deposition. Indeed, it previously been shown that the blockage of autophagy causes α‐syn accumulation.^52^ Taken together, the specific hypoxic treatment regimen in the current study accelerated a vicious cycle of PFF‐induced “p‐α‐syn increase ‐ autophagic flow inhibition ‐ p‐α‐syn aggregates formation.”

**FIGURE 6:**
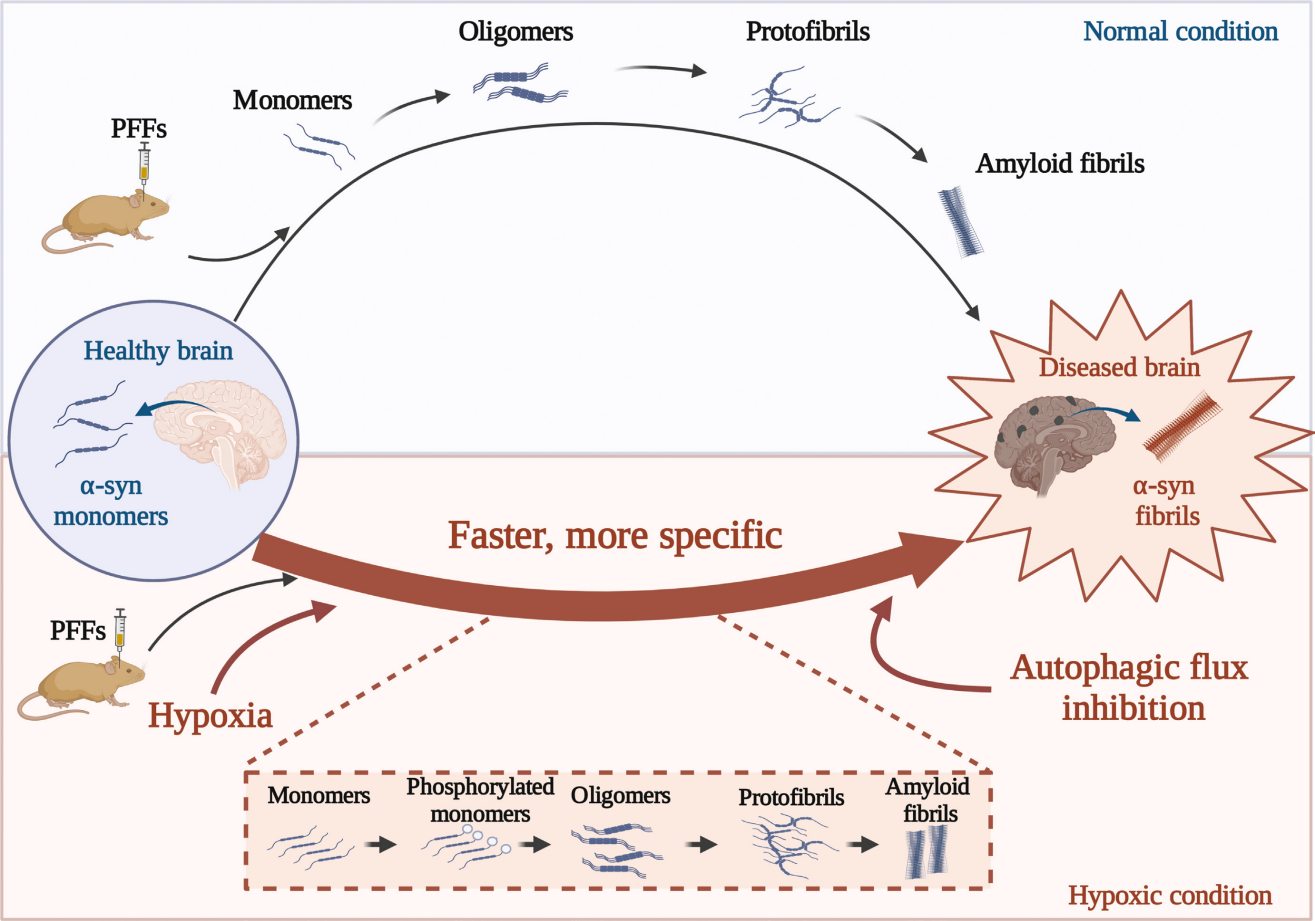
Short‐term hypoxia accelerated pathological α‐syn propagation. Hypoxia combined with stereotactic injection of PFFs results in a rapid modeling pathway. In addition, autophagy inhibition was found in the model mice.

Our study found that hypoxia can accelerate the progression of PD by converting α‐syn into p‐α‐syn, highlighting the important role of hypoxic stress in promoting the pathogenesis of PD (Figure 6). Some studies have proposed that the pathogenesis of PD can be divided into three stages, which are related to triggers, facilitators, and aggravators, respectively.^53^ As a common feature of various PD pathogenesis factors,^36^ hypoxia may be a key mechanism in PD, which still needs further discussion. This study provides reliable evidence for subsequent studies, providing a promising research perspective for the intervention of PD.

## FUNDING INFORMATION

This research was supported by the National Natural Science Foundation of China (Grant number: 32100925, 82027802), the Beijing Nova Program (Grant number: Z211100002121038), the Beijing Hundred Thousand and Ten Thousand Talents Project (Grant number: 2019A36), and the Beijing Municipal Health Commission (Grant number: 303–01–005‐0019).

## CONFLICT OF INTEREST

Dr. Jia Liu is an Editorial Board member of CNS Neuroscience and Therapeutics and a co‐author of this article. To minimize bias, they were excluded from all editorial decision‐making related to the acceptance of this article for publication.

## Supporting information


Appendix S1

## Data Availability

All data generated or analyzed during this study are included in this published article.
