# Supplementary material for: Hypoxic stress accelerates the propagation of pathological alpha‐synuclein and degeneration of dopaminergic neurons
Source: CNS Neurosci Ther. 2022 Dec 13;29(2):544–58. doi: 10.1111/cns.14055 (PMC9873519; doi:10.1111/cns.14055)
Supplement: Supplementary file 1 — Appendix S1 [file CNS-29-544-s001.pdf]

Figure 1 B

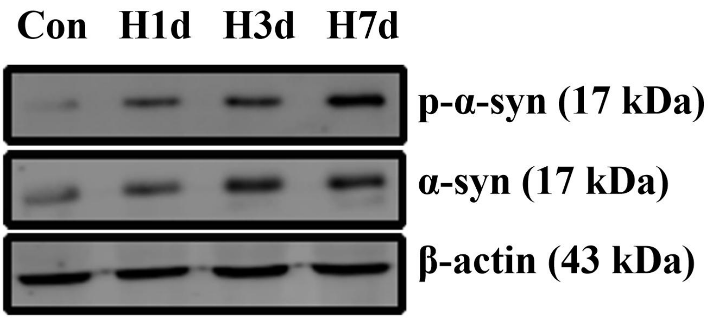

Full unedited gel/blot for Figure 1B

figure 1 B p-α-syn

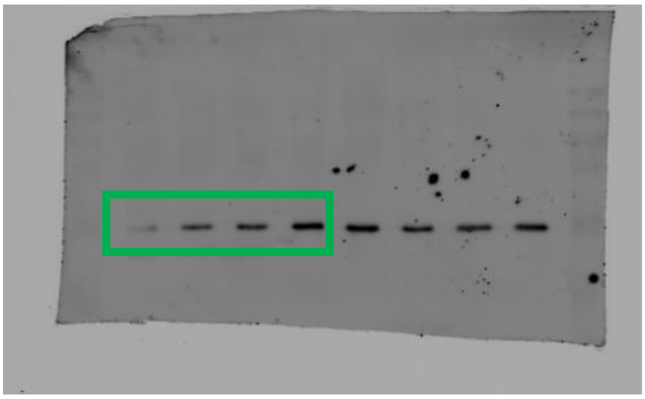

p-α-syn

figure 1 B α-syn

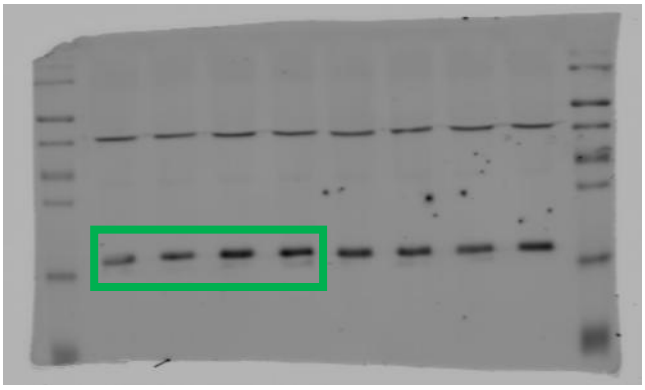

α-syn

figure 1 B β-actin

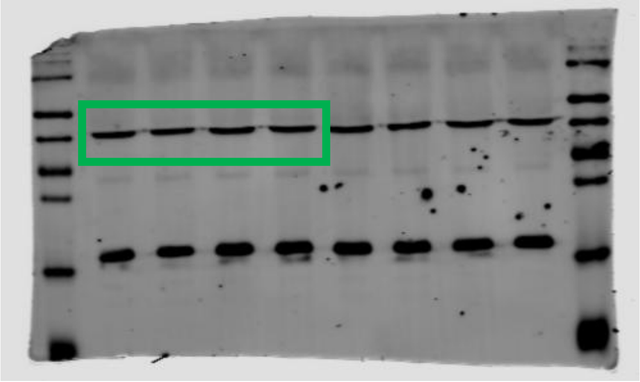

β-actin

Figure 2A

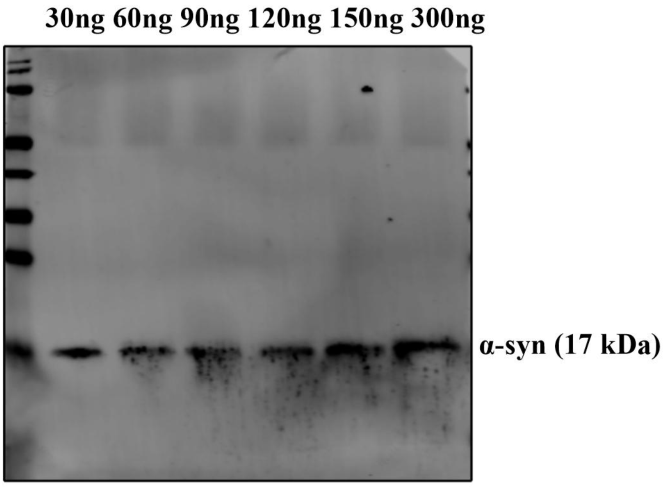

Full unedited gel/blot for Figure 2A

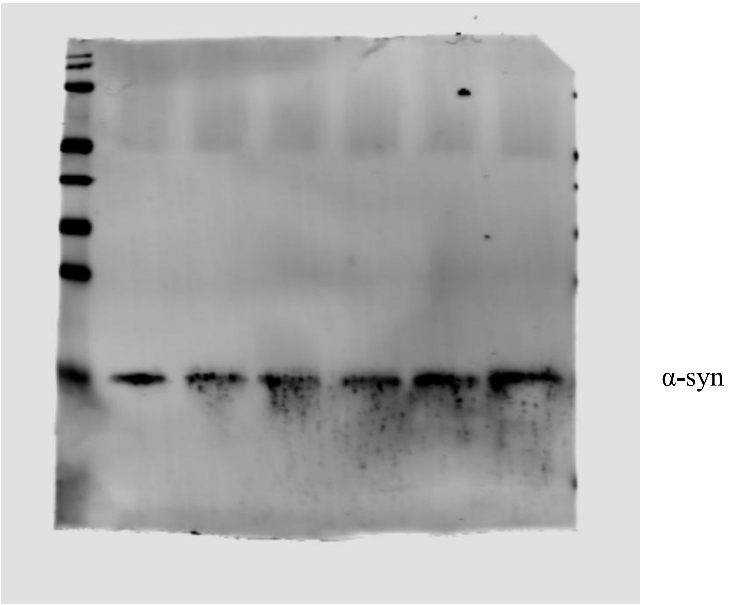

Figure 2B

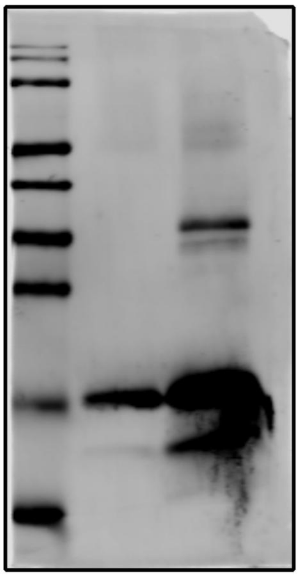

Full unedited gel/blot for Figure 2B

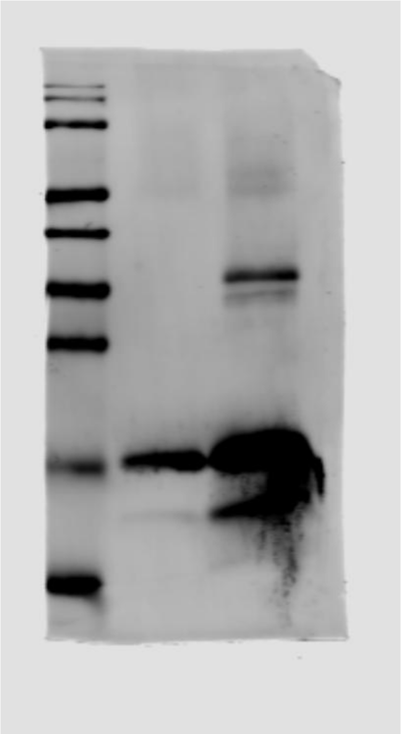

Figure 2D

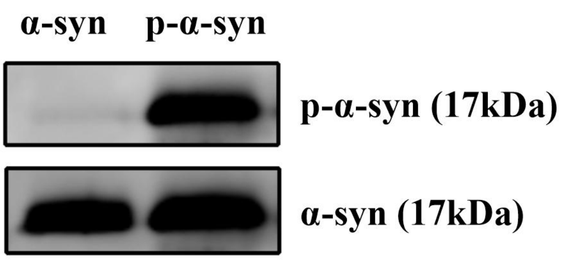

Full unedited gel/blot for Figure 2D

Figure 2D p-α-syn

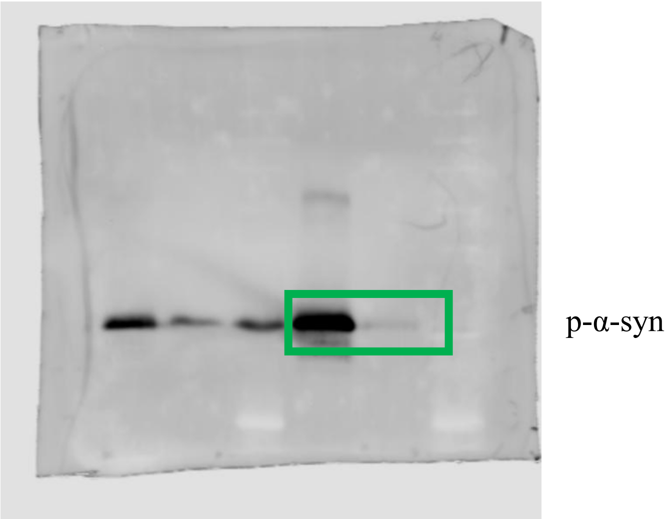

Figure 2D α-syn

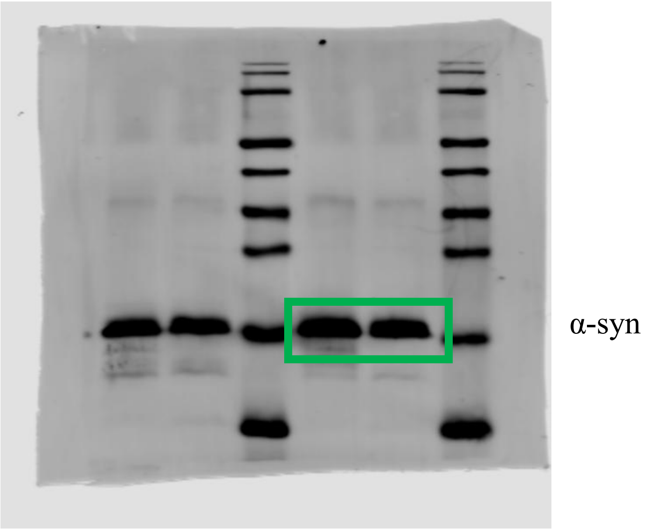

Figure 4B

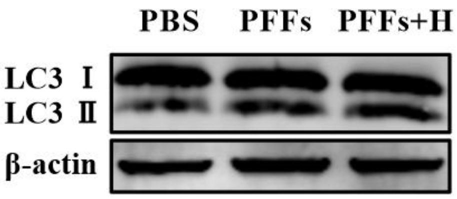

Full unedited gel/blot for Figure 4B

LC3I LC3 II

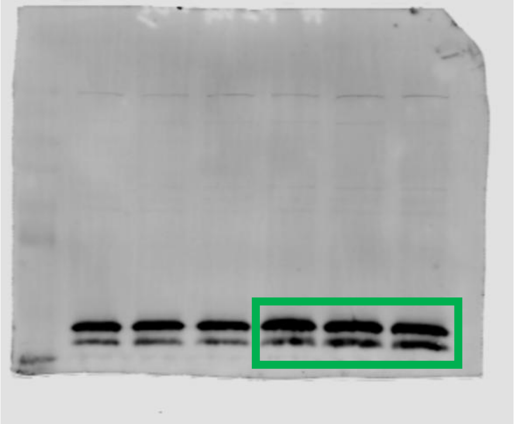

LC3 I
LC3 II

β-actin

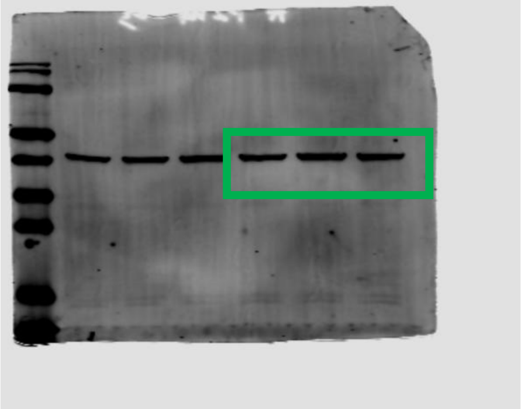

β-actin
